# Ethnic variations in asthma hospital admission, readmission and death: a retrospective, national cohort study of 4.62 million people in Scotland

**DOI:** 10.1186/s12916-015-0546-6

**Published:** 2016-01-12

**Authors:** Aziz Sheikh, Markus F. C. Steiner, Genevieve Cezard, Narinder Bansal, Colin Fischbacher, Colin R. Simpson, Anne Douglas, Raj Bhopal, on behalf of the SHELS researchers

**Affiliations:** Edinburgh Migration, Ethnicity and Health Research Group, Usher Institute of Population Health Sciences and Informatics, The University of Edinburgh, Teviot Place, Edinburgh, EH8 9AG UK; Asthma UK Centre for Applied Research, Usher Institute of Population Health Sciences and Informatics, The University of Edinburgh, Edinburgh, UK; Division of General Internal Medicine and Primary Care and Department of Medicine, Harvard Medical School, Boston, MA USA; Department of Child Health, School of Medicine, University of Aberdeen, Aberdeen, UK; Cardiovascular Epidemiology Unit, The Department of Public Health and Primary Care, University of Cambridge, Cambridge, UK; NHS National Services Scotland, Edinburgh, UK

**Keywords:** Asthma, Death, Epidemiology, Ethnic variations, Hospital admission

## Abstract

**Background:**

Our previous meta-analysis found that South Asians and Blacks in the UK were at a substantially increased risk of hospital admission from asthma. These estimates were, however, derived from pooling data from a limited number of now dated studies, confined to only three very broad ethnic groups (i.e. Whites, South Asians and Blacks) and failed to take account of possible sex-related differences in outcomes within these ethnic groups. We undertook the first study investigating ethnic variations in asthma outcomes across an entire population.

**Methods:**

This retrospective 9-year cohort study linked Scotland’s hospitalisation/death records on asthma to the 2001 census (providing ethnic group). We calculated age, country of birth and Scottish Index of Multiple Deprivation adjusted incident rate ratios (IRRs) for hospitalisation or death by sex for the period May 2001–2010. We calculated hazard ratios (HRs) for asthma readmission and subsequent asthma death.

**Results:**

We were able to link data on 4.62 million people (91.8 % of the Scottish population), yielding over 38 million patient-years of data, 1,845 asthma deaths, 113,795 first asthma admissions, and 107,710 readmissions (40,075 of which were for asthma). There were substantial ethnic variations in the rate of hospitalisation/death in both males and females. When compared to the reference Scottish White population, the highest age-adjusted rates were in Pakistani males (IRR = 1.59; 95 % CI, 1.30–1.94) and females (IRR = 1.50; 95 % CI, 1.06–2.11) and Indian males (IRR = 1.34; 95 % CI, 1.16–1.54), and the lowest were seen in Chinese males (IRR = 0.62; 95 % CI, 0.41–0.94) and females (IRR = 0.49; 95 % CI, 0.39–0.61).

**Conclusion:**

There are very substantial ethnic variations in hospital admission/deaths from asthma in Scotland, with Pakistanis having the worst and Chinese having the best outcomes. Cultural factors, including self-management and health seeking behaviours, and variations in the quality of primary care provision are the most likely explanations for these differences and these now need to be formally investigated.

## Background

Asthma is now one of the most common long-term disorders in the world, with global estimates indicating that at least 300 million people have asthma [1]. The landmark International Study of Asthma and Allergies in Childhood (ISAAC) and the European Community Respiratory Health Survey (ECRHS) studies demonstrated substantial national variations in asthma prevalence, with evidence suggesting that the UK ranks as one of the highest prevalence countries in the world [2, 3]. The recent UK National Review of Asthma Deaths has found persistent problems with poor asthma care and substantial, potentially preventable morbidity and mortality [4]. Investigations conducted within the UK suggest that Scotland has a particularly high morbidity from asthma, for reasons that remain poorly understood [5].

There are, as yet, only a limited number of within-country investigations of ethnic differences in asthma. The majority of this literature comes from the US and this has shown that African-Americans are at increased risk of exacerbations, hospital attendances, near death episodes, and mortality [6–9]. This has led to recent substantial investments ($23 m) to reduce asthma disparities through the Patient-Centered Outcomes Research Institute [10]. A previous systematic review and meta-analysis investigating ethnic variations in asthma outcomes in the UK found that South Asians (odds ratio (OR) = 2.9; 95 % CI, 2.4–3.4) and Blacks (OR = 2.1; 95 % CI, 1.8–2.5) were at substantially increased risk of hospital admission from asthma when compared to White European-origin populations [11]. This synthesis was, however, of only a limited number (n = 13) of now dated studies (undertaken in the 1980–90s), and the summary estimates were therefore only available for three broad ethnic groups, namely: Whites, Blacks and South Asians. The opportunities for exploring heterogeneity within these three groups or indeed investigating differences between males and females in these populations were therefore limited [12].

There is a need for a more contemporaneous, comprehensive investigation into within-country ethnic variations in asthma and asthma outcomes. We report on the first national cohort study to investigate the hypothesis that there are substantial ethnic variations in hospitalisations and deaths from asthma in the Scottish population.

## Methods

### Ethics and permissions

We obtained ethical approval from the Scotland A Research Ethics Committee. We also obtained approvals from Scotland’s Privacy Advisory Committee and the Community Health Index Advisory Group, which are charged with considering the appropriate use of potentially identifiable patient data. Access to data, analyses and release of outputs followed a pre-specified protocol, which included rounding of all count numbers to the nearest five to ensure confidentiality and to prevent disclosure of any potentially identifying data. This included suppression of data if there was any risk that individual patients may be identifiable (n <6). All outputs were reviewed by a National Records of Scotland data disclosure committee.

### Overview of methods and data linkage

**Fig. 1:**
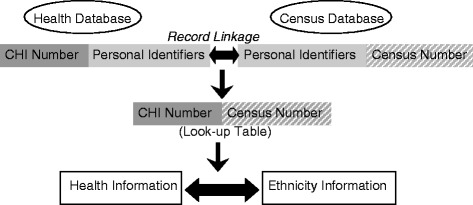
Overview of record linkage process (Community Health Index and census numbers were encrypted)

The methods of the Scottish Health and Ethnicity Linkage Study (SHELS) have previously been described in detail and we therefore confine ourselves here to providing a brief overview of the procedures used [13]. In summary, we linked National Health Service (NHS) Scotland’s hospital discharge and death records (SMR01) to the 2001 census using probabilistic linkage methods creating a unique link between the encrypted Community Health Index and census number, thereby creating a national retrospective cohort. An overview of the linkage techniques is shown in Fig. 1. All personal identifiers were then removed from the linked dataset.

### Ethnicity data

Ethnic categories were derived from the 14 groups in the 2001 Scottish Census (Box 1) and reported following the principles of the census (including capitalisation of ethnic group labels) [14]; when discussing the work of others, however, we used the labels assigned by authors in their publications. Given small populations, we combined Bangladeshis with Other South Asians and the Caribbean, African and Black Scottish or Other Black as an African origin group, thus giving rise to the nine categories of ethnicity that were studied and reported on.

### Asthma outcomes

We had three outcomes of interest: (1) first hospitalisation or death from asthma following inception of the cohort in May 2001, which was our primary outcome and is henceforth referred to as ‘first asthma events’ (group 1); (2) asthma readmission among survivors of group 1; and (3) asthma death among survivors of group 1. We identified hospitalisations and deaths for asthma during the period May 2001–April 2010 using relevant International Classification of Disease codes (i.e. ICD-9: 493 and 493.9, and ICD-10: J45 and J46). To take account of the chronic nature of asthma, we did not exclude those with admissions before May 2001 [15]. Our ‘first asthma events’ were therefore initial episodes within the cohort study, which may or may not correspond with the first ever episodes of hospitalisation.

### Demographic and socioeconomic factors

Census data included age, sex, Scottish Index of Multiple Deprivation (SIMD), and country of birth (CoB). SIMD is an area-based socioeconomic measure that uses 38 indicators across nine domains including health to rank areas based on full postcode [16]. The SIMD is known to be associated with health outcomes whether it includes, or excludes, the health domains.

### Analysis

Our detailed analysis plan specified that, because of concerns around small numbers and the associated risk of disclosure, analysis of hospitalisation and death would only be separately undertaken if deaths were at least 20 % of the total numbers. We also pre-specified that if this was not the case, the first of either of these two events would be included, thereby ensuring that we included community deaths.

Using Poisson regression models with robust variance we calculated age-adjusted rates of first asthma events (i.e. outcome (1) specified above) per 100,000 person years and incidence rate ratios (IRR) with 95 % confidence intervals (CI) by sex, this being undertaken because of the known sex variations in asthma epidemiology and outcomes. The person-years-at-risk denominators were calculated after censoring for deaths, asthma-related hospitalisations and transfers of patients out of NHS Scotland (i.e. to other parts of the UK), as appropriate.

The age-adjusted sex-stratified analysis was the primary analysis. We then examined the effect of adjustment by socioeconomic variables and CoB separately and in combination. We explored the association between eight measures of socioeconomic status (i.e. individual educational level, household educational level, individual National Statistics Socioeconomic Classification, household National Statistics Socioeconomic Classification, SIMD quintile, car ownership, household tenure, and economic activity) and health outcomes following our published approach, and we selected SIMD (quintiles), which was available for everyone; in contrast, other relevant variables were not collected for younger and older age groups [17]. Our analysis used the quintile of socioeconomic deprivation. We adjusted for birth within or outside the UK to explore whether this altered IRRs (there were not enough events for stratified analysis by CoB and ethnicity).

We undertook survival analysis and calculated hazard ratios (HRs) for death or readmission with asthma following hospitalisation (outcomes (2) and (3) as specified above, censoring for death) using Cox proportional hazard models, checking the proportional hazards assumption with the graphical method using log-log (ln{-ln(survival)}) plots. Analysis was undertaken using SAS v9.3 (SAS Institute Inc., Cary, North Carolina, USA).

## Results

### Overview of the cohort

**Fig. 2:**
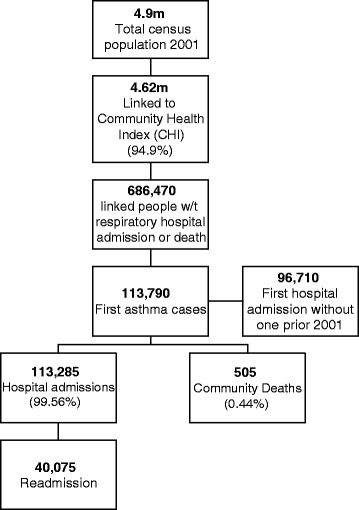
Study flow diagram

Scotland had an enumerated population of 4.9 million on census day (29 April 2001); 52 % of the population was female and 89 % was White Scottish (Table 1). We were able to link data on 4.62 million individuals (94.9 % of the eligible population), yielding 38,216,530 patient-years of data over the 9-year study period (Fig. 2). We successfully linked data on at least 85 % of people from each ethnic group of interest (Tables 1 and 2).

**Table 1:** Baseline characteristics of study population

	Census		First asthma admissions and deaths		Male		Female		% Female in each group	First asthma admissions incidence from 2001	
	n	%	n	%	n	%	n	%	%	n	%
White Scottish	4,088,125	88.6	102,285	89.9	41,955	89.8	60,330	89.9	59.0 %	86,930	89.9
Other White British	334,985	7.3	7,555	6.6	3,005	6.4	4,550	6.8	60.2 %	6,515	6.7
White Irish	43,505	0.9	985	0.9	390	0.8	590	0.9	60.2 %	825	0.9
Other White	65,655	1.4	1,030	0.9	410	0.9	620	0.9	60.2 %	895	0.9
Any mixed background	11,110	0.2	265	0.2	115	0.2	150	0.2	56.6 %	220	0.2
Indian	12,335	0.3	305	0.3	160	0.3	150	0.2	48.4 %	225	0.2
Pakistani	25,630	0.6	840	0.7	415	0.9	420	0.6	50.3 %	655	0.7
Other South Asian	6,510	0.1	155	0.1	80	0.2	75	0.1	48.4 %	135	0.1
African origin	6,335	0.1	120	0.1	50	0.1	70	0.1	58.3 %	105	0.1
Chinese	13,205	0.3	145	0.1	75	0.2	70	0.1	48.3 %	120	0.1
All other ethnic groups	7,715	0.2	105	0.1	50	0.1	55	0.1	52.4 %	90	0.1
Total	4,615,105		113,790		46,710		67,080		59.0 %	96,710

**Table 2:** Further baseline characteristics of study population

		Age groups				Age, years	Country of birth		SIMD quintiles				
	n	<20	20 to <40	40 to <60	60+	Mean	UK born	Outside UK	1	2	3	4	5
White Scottish	102,285	23.7 %	21.6 %	26.9 %	27.8 %	41.7	99.1 %	0.9 %	26.2 %	23.6 %	20.5 %	16.7 %	13.0 %
Other White British	7,555	11.2 %	21.0 %	34.0 %	33.9 %	48.8	95.8 %	4.2 %	12.0 %	16.3 %	26.8 %	25.7 %	19.1 %
White Irish	985	7.1 %	17.3 %	32.5 %	43.1 %	53.6	99.0 %	1.0 %	27.9 %	24.9 %	19.8 %	14.2 %	13.2 %
Other White	1,030	18.9 %	22.8 %	27.7 %	30.6 %	44.3	36.4 %	63.6 %	16.6 %	21.5 %	22.0 %	19.5 %	20.5 %
Any mixed background	265	54.7 %	17.0 %	17.0 %	11.3 %	25.6	83.0 %	17.0 %	27.8 %	18.5 %	16.7 %	18.5 %	18.5 %
Indian	305	27.9 %	26.2 %	24.6 %	21.3 %	36.9	51.6 %	48.4 %	13.1 %	21.3 %	11.5 %	18.0 %	36.1 %
Pakistani	840	33.5 %	21.6 %	28.1 %	16.8 %	33.6	46.4 %	53.6 %	17.4 %	28.7 %	13.8 %	17.4 %	22.8 %
Other South Asian	155	32.3 %	19.4 %	32.3 %	16.1 %	34.8	51.6 %	48.4 %	22.6 %	22.6 %	12.9 %	19.4 %	22.6 %
African origin	120	41.7 %	25.0 %	25.0 %	8.3 %	30.9	62.5 %	37.5 %	29.2 %	20.8 %	16.7 %	20.8 %	12.5 %
Chinese	145	48.3 %	17.2 %	20.7 %	13.8 %	29.5	55.2 %	44.8 %	20.7 %	13.8 %	10.3 %	20.7 %	34.5 %
All Other Ethnic group	105	33.3 %	28.6 %	28.6 %	9.5 %	33.8	33.3 %	66.7 %	19.0 %	19.0 %	19.0 %	14.3 %	28.6 %
Total	113,790	22.9 %	21.5 %	27.4 %	28.2 %	42.1	97.6 %	2.4 %	25.1 %	23.1 %	20.8 %	17.3 %	13.7 %

### First asthma events

**Fig. 3:**
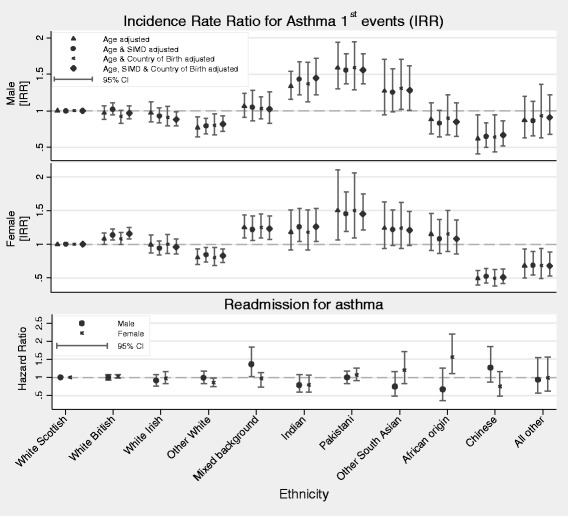
Summary data for ethnic variations in incidence rate ratios of first asthma events and hazard ratios of readmissions

There was a total of 113,790 first asthma admissions (n = 67,080; 59 % in females) and 505 deaths with asthma diagnosis and no prior hospital admission for asthma (59 % in females). There were substantial ethnic variations in first asthma events in both males and females (Fig. 3). Age-adjusted incidence rates for a first asthma event in the total population were 256.6 (95 % CI, 208.1–316.3) per 100,000 person years in males and 338.1 (288.7–395.9) in females.

When compared to the White Scottish reference population, the highest age-adjusted rates were in Pakistani males (IRR = 1.59; 95 % CI, 1.30–1.94) and females (IRR = 1.50; 95 % CI, 1.06–2.11) and Indian males (IRR = 1.34; 95 % CI, 1.16–1.54), and the lowest were seen in Chinese males (IRR = 0.62; 95 % CI, 0.41–0.94) and females (IRR = 0.49; 95 % CI, 0.39–0.61) followed by ‘Other White’ males (IRR = 0.77; 95 % CI, 0.64–0.92) and females (IRR = 0.81; 95 % CI, 0.70–0.93). Further adjustment for SIMD and CoB did not substantially modify the observed patterns (Table 3).

**Table 3:** Incidence rates, incidence rate ratios (IRRs), and hazard ratio estimates per 100,000 population per year (PY) for first asthma hospital admission or death. IRRs are age adjusted and rate ratios are subsequently Scottish Index for Multiple Deprivation (SIMD) and country of birth (COB) adjusted, with 95 % confidence intervals (CIs), by ethnic group, for the population in Scotland

Ethnic group	First asthma event^a^	PY at risk^b^	Age adjusted incidence rates (per 100,000 PY) and 95 % CIs^c^	Age-adjusted IRRs^d^	Age- and SIMD-adjusted IRR^d^	Age- and CoB-adjusted IRR^d^	Age-, SIMD- and CoB-adjusted IRR^d^
				IRR and 95 % CI	IRR and 95 % CI	IRR and 95 % CI	IRR and 95 % CI
Males							
White Scottish	41,955	16,325,930	257.0	1.00 (. .)	1.00 (. .)	1.00 (. .)	1.00 (. .)
Other White British	3,005	1,244,330	249.7 (227.7–276.9)	0.97 (0.88–1.07)	1.02 (0.94–1.11)	0.92 (0.83–1.01)	0.97 (0.89–1.069)
White Irish	390	160,090	250.2 (217.4–287.8)	0.97 (0.85–1.12)	0.93 (0.84–1.04)	0.91 (0.79–1.06)	0.88 (0.79–0.99)
Other White	410	223,900	196.5 (164.8–238.4)	0.78 (0.64–0.92)	0.79 (0.69–0.90)	0.80 (0.67–0.96)	0.82 (0.72–0.93)
Any mixed background	115	42,835	273.2 (235.0–320.4)	1.06 (0.91–1.24)	1.05 (0.86–1.28)	1.03 (0.89–1.19)	1.02 (0.83–1.26)
Indian	160	50,445	344.5 (300.3–397.8)	1.34 (1.16–1.54)	1.43 (1.22–1.67)	1.37 (1.12–1.66)	1.45 (1.22–1.72)
Pakistani	420	109,375	408.1 (336.3–501.8)	1.59 (1.30–1.94)	1.56 (1.37–1.78)	1.59 (1.29–1.95)	1.56 (1.37–1.78)
Other South Asian	80	27,265	327.3 (244.6–441.3)	1.27 (0.94–1.71)	1.25 (0.99–1.57)	1.31 (1.02–1.71)	1.28 (1.01–1.62)
African Origin	50	25,020	225.3 (178.4–286.6)	0.88 (0.69–1.11)	0.83 (0.64–1.01)	0.90 (0.67–1.22)	0.85 (0.65–1.11)
Chinese	75	52,825	159.1 (105.6–241.5)	0.62 (0.41–0.94)	0.65 (050–0.84)	0.64 (0.44–0.94)	0.67 (0.52–0.86)
All Other Ethnic Group	50	25,650	223.5 (162.1–310.6)	0.87 (0.63–1.20)	0.86 (0.66–1.13)	0.93 (0.63–1.36)	0.91 (0.68–1.22)
Ethnic group	First asthma event^a^	PY at risk^b^	Age-adjusted incidence rates (for 100,000 PY)^c^	Age-adjusted IRR^d^	Age- and SIMD-adjusted IRR^d^	Age- and CoB-adjusted IRR^d^	Age-, SIMD- and CoB-adjusted IRR^d^
				IRR and 95 % CI	IRR and 95 % CI	IRR and 95 % CI	IRR and 95 % CI
Females							
White Scottish	60,330	17,830,550	338.3	1.00 (. .)	1.00 (. .)	1.00 (. .)	1.00 (. .)
Other White British	4,550	1,334,530	365.4 (339.3–397.6)	1.08 (1.00–1.17)	1.14 (1.06–1.23)	1.08 (1.00–1.18)	1.16 (1.08–1.25)
White Irish	590	178,670	335.9 (295.7–387.0)	0.99 (0.87–1.14)	0.94 (0.84–1.05)	1.00 (0.86–1.15)	0.96 (0.85–1.08)
Other White	620	259,410	271.6 (237.3–315.5)	0.80 (0.70–0.93)	0.84 (0.74–0.95)	0.80 (0.68–0.95)	0.83 (0.73–0.93)
Any mixed background	150	46,190	424.1 (369.5–489.7)	1.25 (1.09–1.44)	1.22 (1.06–1.42)	1.25 (1.09–1.45)	1.23 (1.07–1.42)
Indian	150	45,810	398.1 (311.2–512.3)	1.18 (0.92–1.51)	1.26 (1.04–1.53)	1.18 (0.92–1.51)	1.26 (1.04–1.53)
Pakistani	420	107,485	506.1 (361.1–718.0)	1.50 (1.06–2.11)	1.45 (1.19–1.78)	1.50 (1.09–2.06)	1.45 (1.21–1.75)
Other South Asian	75	22,205	418.9 (318.9–553.6)	1.24 (0.94–1.63)	1.22 (0.98–1.51)	1.24 (0.94. 1.62)	1.21 (0.98–1.49)
African Origin	70	22,215	390.4 (309.7–495.0)	1.15 (0.91–1.46)	1.08 (0.86–1.37)	1.15 (0.89–1.50)	1.08 (0.85–1.36)
Chinese	70	52,660	165.3 (132.5–207.5)	0.49 (0.39–0.61)	0.52 (0.42–0.64)	0.49 (0.38–0.62)	0.51 (0.41–0.63)
All Other Ethnic Group	55	29,145	230.6 (170.4–314.1)	0.68 (0.50–0.93)	0.69 (0.54–0.89)	0.68 (0.49–0.94)	0.68 (0.53–0.88)

^a^First event within the period 1st May 2001 to 30th April 2010, with no look back previous to this period

^b^Over 9-year period between 1st May 2001 and 30th April 2010

^c^Poisson rates are derived with age adjustment using the true White Scottish rate

^d^Incidence rate ratios are calculated using Poisson regression models with robust variance. The White Scottish population at the 2001 census is the reference population

### Asthma readmissions among survivors of a first asthma event

There were 107,710 readmissions in the cohort; 40,075 of these readmissions were for asthma (37 % of all readmissions for asthma), with the majority of these occurring in females (n = 24,660; 61.5 %). Increased rates of readmission were seen in Any Mixed Background males (HR = 1.37; 95 % CI, 1.02–1.83) and African Origin females (HR = 1.56, 95 % CI, 1.11–2.20). Other than this, no clear patterns were apparent, this possibly reflecting the relatively small number of readmissions within most of the minority ethnic groups and the associated imprecision of the HR estimates obtained (Table 4).

**Table 4:** Hazard ratios (HRs) adjusted for age, relative to White Scottish of hospital readmission for asthma following a first hospital admission of asthma during the study period (i.e. 1/5/2001–31/4/2010) in men and women

	Males		Females	
Ethnic group	Number of readmissions	Overall readmission	Number of readmissions	Overall readmission
		HR and 95 % CI		HR and 95 % CI
White Scottish	13,795	1.00 (. .)	22,115	1.00 (. .)
Other White British	1,040	1.00 (0.93–1.07)	1,740	1.03 (0.98–1.08)
White Irish	130	0.91 (0.76–1.08)	225	0.97 (0.84–1.16)
Other White	140	0.99 (0.83–1.18)	200	0.86 (0.74–0.99)
Any mixed background	45	1.37 (1.02–1.83)	50	0.97 (0.73–1.13)
Indian	45	0.79 (0.59–1.07)	50	0.79 (0.60–1.06)
Pakistani	145	1.00 (0.84–1.18)	170	1.07 (0.91–1.25)
Other South Asian	20	0.75 (0.49–1.17)	30	1.20 (0.84–1.71)
African Origin	10	0.68 (0.36–1.26)	35	1.56 (1.11–2.20)
Chinese	25	1.27 (0.87–1.85)	20	0.76 (0.49–1.16)
All Other Ethnic Group	15	0.93 (0.56–1.55)	20	0.99 (0.62–1.57)

### Asthma deaths among survivors of a first asthma event

In total, there were 1,340 asthma deaths recorded in those who survived the first asthma event. As death was an infrequent occurrence (0.4 % of first asthma events) data on survival were suppressed for most ethnic groups because of the possible risk of disclosure. The available data on Other White British males (HR = 0.69; 95 % CI, 0.45–1.06) and Other White British (HR = 0.93; 95 % CI, 0.70–1.23), White Irish (HR = 1.08; 95 % CI, 0.58–2.01) and Other White (HR = 1.27; 95 % CI, 0.63–2.55) females was imprecisely estimated.

## Discussion

### Statement of principal findings

This national investigation into risk of poor asthma outcomes has found substantial ethnic variations in the rates of hospital admission and death between ethnic groups in Scotland. When compared to the reference White Scottish population, people of South Asian descent (i.e. Pakistani, Indian and Other South Asians) had 20–50 % increased rates of hospitalisation from asthma, whereas people of Chinese origin had 30–40 % lower rates. Comparing the Chinese with the Pakistani populations revealed a striking 2–3-fold variation in rates of admission. However, despite accruing over 38 million person-years of data during the 9-year study period, we obtained only imprecise estimates of the rates of death and readmission in survivors, this reflecting the relative infrequency of these outcomes in many of the populations studied.

### Strengths and limitations

This is, as far as we are aware, the first national investigation into within-country ethnic variations in asthma outcomes and it has provided some of the first estimates of asthma outcomes in a number of sub-sections of the UK population (e.g. White Irish and Chinese) [11]. Additional key strengths of this work include the substantial number of patient-years of data available, which offered the possibility to study a large number of hospitalisations for asthma – the main focus of our study. Through linking to the 2001 Census we were able to simultaneously adjust for age, CoB and socioeconomic status [18]. The decision to stratify reporting by sex was important given the marked overall increased rate of admission, readmission and mortality in females. Furthermore, in keeping with best practice, our analysis strategy was written and agreed prior to data analysis.

Nonetheless, we acknowledge limitations, including the fact that we were not able to link data on the entire Scottish population, differential rates in linkage between ethnic groups which may have introduced bias, the fact that we were not fully able to take into account emigration from Scotland, that event numbers in some non-White ethnic groups were small (particularly limiting for survival analysis), and that, because analysis of data on small groups potentially risked disclosing identity, we were not able to report on mortality data for the majority of the groups studied. Missing data may also have introduced the possibility of bias. It is impossible to know how much missing data there was overall, but we believe that this is likely to be small. This missing data could have arisen for three possible reasons: (1) those who did not participate in the census (estimated <5 %); (2) those for whom it was not possible to link census and hospitalisation and mortality data (estimated <5 % at date of census, and increases with length of time from the census due to immigration and births); and (3) those admitted into private hospitals that did not then convey this information to the Information Services Division of NHS National Services Scotland (<1 %). We attempted to censor those who migrated, but it was not possible to identify the likely small proportion of people who were not registered with NHS Scotland who emigrated or those who were registered with NHS Scotland but who emigrated outside of the UK. Finally, we were only able to adjust for a limited number of potential confounders in the relationship between ethnicity and asthma outcome and, as discussed below, future work needs to consider the impact of wider organisational, care-related and behavioural factors which may help to explain these variations.

### Interpreting the findings in the context of the wider literature and implications for future research

These findings confirm the poor outcomes previously noted for UK South Asians, but were not as marked, possibly reflecting the relatively high rate of hospitalisation in the reference Scottish White population and/or the reduced risk of bias resulting from the fact that we were able to study the entire population [11]. These data also reveal that not all ethnic minority groups experience poor outcomes when compared with the reference population. In particular, the rate of asthma admission in Chinese-origin males and females were markedly lower than that seen in the White Scottish population. Given that good outcomes in the Scottish Chinese population have also been noted for a range of other long-term conditions such as heart failure [19], there is a need to investigate whether this is due to Chinese populations having comparatively better health, lower use of health services, or a combination of both of these factors.

There is now a need to understand the reasons underpinning these striking variations in outcomes. Possibilities include biological differences in disease severity, the impact of migration (although we did not find any effect of adjusting for CoB) [18], and the greater prevalence of atopy noted in South Asian children in the UK [20]. Another potentially important explanatory consideration is differences in over-crowding with the associated risk of admissions triggered by viral upper respiratory tract infections. Differences in health professional management including access to and quality of primary care (such as vaccination rates, prescribing and use of supported self-management plans) [21] and cultural factors, including differences in personal beliefs, self-management (in particular, concordance with preventive therapies) and health-seeking behaviour, may also be important contributory factors [22]. US data have, for example, shown marked variations in the quality of ambulatory and emergency department asthma care, but comparable hospital-based care [23]. There is also a need to consider the possibility of systematic biases through investigating the impact of differential rates of linkage and emigration between ethnic groups [24].

Data on a number of potential explanatory variables are available in general practitioner records, and our plan is therefore to link the SHELS cohort with primary care data across Scotland. We have undertaken a feasibility pilot (data being prepared for publication) and other primary care data extractions [25], which shows that this is technically possible and that relevant data on, for example, smoking, vaccination and prescribing patterns can be obtained for future studies. A key priority for future work will be to investigate to what extent these variations in outcome can be explained by differences in lifestyle factors, health-seeking behaviours and/or the quality of primary care [21, 22, 26, 27]. Future work should also explore the possibility of interactions between ethnicity and socioeconomic status.

## Conclusions

This study has provided compelling evidence of important national variations in rates of asthma hospital admissions/deaths across ethnic groups in Scotland. It seems likely that the majority of these variations are amenable to lifestyle and/or medical intervention in primary care, and a key near-term target should therefore be to reduce the risk of hospitalisation/death for all ethnic groups, but especially in Pakistani males and females and Indian males in Scotland.

### Availability of supporting data

We do not have permission to share the underlying dataset as, due to security considerations, this needs to be analysed within the confines of a safe haven. Researchers should contact the principal investigator if they wish to explore the possibility of collaborative work.

## Box 1: Scotland 2001 Census ethnic groups

White Scottish

Other White British

White Irish

Other White

Any mixed background

Indian

Pakistani

Bangladeshi

Other South Asian

Caribbean

African

Black Scottish or Other Black

Chinese

Other ethnic group

## Abbreviations

CI: Confidence interval
CoB: Country of birth
HR: Hazard ratio
IRR: Incidence rate ratio
NHS: National Health Service
OR: Odds ratio
PY: Person years
SHELS: Scottish Health and Ethnicity Linkage Study
SIMD: Scottish Index of Multiple Deprivation
